# Patient-reported quality indicators to evaluate physiotherapy care for hip and/or knee osteoarthritis- development and evaluation of the QUIPA tool

**DOI:** 10.1186/s12891-020-03221-5

**Published:** 2020-04-01

**Authors:** Pek Ling Teo, Rana S. Hinman, Thorlene Egerton, Krysia S. Dziedzic, Jessica Kasza, Kim L. Bennell

**Affiliations:** 1 Centre for Health, Exercise and Sports Medicine, Department of Physiotherapy, School of Health Sciences, The University of Melbourne, Melbourne, Victoria 3010 Australia; 2 School of Primary Community and Social Care, Keele University, Staffordshire, UK; 3 Department of Epidemiology and Preventive Medicine, Monash University, Melbourne, Australia

**Keywords:** Quality indicators, Physiotherapy, Hip, Knee, Osteoarthritis, Patient-reported, Reliability, Validity, Quality of care

## Abstract

**Background:**

There is no physiotherapy-specific quality indicator tool available to evaluate physiotherapy care for people with hip and/or knee osteoarthritis (OA). This study aimed to develop a patient-reported quality indicator tool (QUIPA) for physiotherapy management of hip and knee OA and to assess its reliability and validity.

**Methods:**

To develop the QUIPA tool, quality indicators were initially developed based on clinical guideline recommendations most relevant to physiotherapy practice and those of an existing generic OA quality indicator tool. Draft items were then further refined using patient focus groups. Test-retest reliability, construct validity (hypothesis testing) and criterion validity were then evaluated. Sixty-five people with hip and/or knee OA attended a single physiotherapy consultation and completed the QUIPA tool one, twelve- and thirteen-weeks after. Physiotherapists (*n* = 9) completed the tool post-consultation. Patient test-retest reliability was assessed between weeks twelve and thirteen. Construct validity was assessed with three predefined hypotheses and criterion validity was based on agreement between physiotherapists and participants at week one.

**Results:**

A draft list of 23 clinical guideline recommendations most relevant to physiotherapy was developed. Following feedback from three patient focus groups, the final QUIPA tool contained 18 items (three subscales) expressed in lay language. The test-retest reliability estimates (Cohen’s Kappa) for single items ranged from 0.30–0.83 with observed agreement of 64–94%. The intraclass correlation coefficient (ICC) and 95% confidence interval (CI) for the Assessment and Management Planning subscale was 0.70 (0.54, 0.81), Core Recommended Treatments subscale was 0.84 (0.75, 0.90), Adjunctive Treatments subscale was 0.70 (0.39, 0.87) and for the total QUIPA score was 0.80 (0.69, 0.88). All predefined hypotheses regarding construct validity were confirmed. However, agreement between physiotherapists and participants for single items showed large measurement error (Cohen’s Kappa estimates ranged from − 0.04-0.59) with the ICC (95% CI) for the total score being 0.11 (− 0.14, 0.34).

**Conclusions:**

The QUIPA tool showed acceptable test-retest reliability for subscales and total score but inadequate reliability for individual items. Construct validity was confirmed but criterion validity for individual items, subscales and the total score was inadequate. Further research is needed to refine the QUIPA tool to improve its clinimetric properties before implementation.

## Background

Osteoarthritis (OA) is a leading cause of joint pain and disability worldwide [1] with the overall prevalence of hip and knee OA in the adult population approximately 11 and 24% respectively [2]. Osteoarthritis costs Australia’s economy $22 billion annually, and the burden of OA is expected to rise due to the ageing population and obesity [3, 4]. Physiotherapists play an integral role in providing non-pharmacological management for OA. A systematic review of patients’ perceived health service needs for OA showed that patients generally perceive physiotherapists to be important to assist them in managing their condition and prescribing exercises [5].

Despite international OA guidelines recommending exercise and weight loss [1, 6, 7] as first line treatments for OA, their uptake is suboptimal in physiotherapy practice [8–11]. Quality indicators (QIs) can be used to assess physiotherapists’ adherence to clinical guidelines recommendations and are accepted tools for assessing OA care [12–14]. They represent minimal acceptable standards of practice [15, 16] and are typically developed via consensus techniques [17, 18]. Quality indicators can be assessed by auditing medical records [18] however, these do not always include information pertaining to quality of care. Self-reporting by health professionals is another method but may introduce bias. To overcome limitations of these methods, patient-reported QIs are an alternative option to assess quality of OA care. Patient involvement in quality assessment is also valuable to enhance quality and relevance of research [19] as well as to promote patient-centred care, one of the six pillars of high quality care [20].

A systematic review conducted in 2013 identified QIs from 32 papers pertaining to non-pharmacological, pharmacological and surgical management for OA [13] but found only one study [21] (from Norway) that developed QIs in a patient-reported format. Blackburn and colleagues [22] later developed a similar QI questionnaire in the United Kingdom (UK) by including items from the Norwegian questionnaire. The UK-QI questionnaire was subsequently used by patients across several European countries to assess the care they received from a range of health professionals for their OA management [23, 24]. However, the UK questionnaire was not tailored to specifically evaluate physiotherapy care. In the Netherlands in 2016, a set of QIs for physiotherapy management in hip and knee OA was established using a Delphi technique [25] but was not developed into a patient-reported tool. Furthermore, the QIs were based on older clinical guidelines from 2011 [26] and were not developed with an international perspective given they only recruited a national group of experts for their Delphi panel.

Given the lack of specific patient-reported QIs to assess physiotherapy care for hip and/or knee OA, this study aimed to develop a patient-reported QI tool and to evaluate its clinimetric properties. It is vital to establish the validity and reliability of QI tools if the results are to accurately reflect physiotherapy practice and/or be used to guide decision-making to improve clinical services [27]. These measurement criteria are prerequisite for any quality measure and should be established prior to implementation of the QIs [16, 18].

## Methods

### Phase 1: tool development

We used two sequential stages to develop the Quality Indicators for Physiotherapy Management of Hip and Knee Osteoarthritis (QUIPA) tool: 1) drafting of patient-reported QIs based on clinical guideline recommendations most relevant to physiotherapy practice identified from a recent consensus study [28] and the UK-QI questionnaire [22] and 2) refinement of the language and format of the QUIPA tool to ensure it was consumer friendly.

The research members involved in this study included physiotherapists who are also experts in OA research (KB, RH, TE, KD) and QI development and implementation (KD). KD has extensive experience in QI development and use for implementation of National Institute for Health and Care Excellence Quality Standards through clinical tools and patient questionnaires. KD was involved in the UK-QI study [13] which included patient and public involvement and engagement, as experts by experience.

### Stage 1 – drafting of patient-reported quality indicators for physiotherapy care

Draft QIs were derived from a final list of clinical guideline recommendations for hip and/or knee OA proposed by a recent consensus study as being most relevant to physiotherapy care [28]. The study first extracted recommendations from two high-quality clinical guidelines [1, 29] and then included a panel of 62 international physiotherapists to complete an online modified-Delphi survey, followed by a priority-ranking exercise in order to identify and rank recommendations most relevant for physiotherapy practice. The final 30 recommendations were then synthesized and grouped by content area to convey a physiotherapy management for hip and/or knee OA. A conceptual model based on the results of the study [28] was used when developing the QUIPA tool. The four main content areas of the final recommendations were condensed to form the three subscales of the QUIPA tool. We aimed to develop a QI relevant to each of the 30 recommendations on the final list, whilst minimising redundancy across items. Thus, where recommendations were similar, we only developed a QI based on the highest ranked recommendation [28]. We did not develop a QI for recommendations if it was deemed by the research experts as difficult to assess in a physiotherapy consultation; captured in another individual QI; related to a health service program instead of an individual treatment or unable to be executed by a physiotherapist (e.g. referring patients for joint surgery). Where the recommendations overlapped with those in the UK-QI questionnaire [22], we utilised similar phrasing as the UK-QI questionnaire because it had been through a rigorous development process, involved patient participation and was based on the most recent QIs, both from the Norwegian patient-reported QI questionnaire [21] and the systematic review in 2013 [13]. Although the Norwegian team has since revised and validated their QI questionnaire [30], it contains similar QIs to that of the previous version. The first draft of the QUIPA tool is attached in Additional file 1.

### Stage 2 – refinement of the language and format of the QUIPA tool

#### Patient and public involvement

A convenience sample of 15 people with hip and/or knee OA living in Melbourne, Australia were recruited from our research database and via Facebook to participate in one of three face-to-face focus groups to further refine the QUIPA tool. Inclusion criteria were: i) aged 45 years or above, ii) being told they had OA in their hip and/or knee by a health professional, iii) saw a physiotherapist for their hip and/or knee OA over the last 3 months, and iv) able to attend the University for allocated session date/time. Ethical approval was granted by the School of Health Sciences Human Ethics Advisory Group, University of Melbourne (Ethics Application 1,750,532).

Each focus group session ran for 90 min and was moderated by a research team member and an assistant. Sessions were audio-recorded. Participants firstly completed a questionnaire about demographics as well as hip/knee pain and function. They were then presented with the draft QUIPA tool and asked to explain what they understood each QI item meant to ensure consistency with its original intent, a technique known as cognitive debriefing [31]. They were also asked to comment on wording clarity. The QUIPA tool was projected onto a presentation screen to allow the research assistant to alter the wording of the QIs in real time during the group session. Participants were also asked to comment on the appropriateness of the tool response scale and its overall format and layout [22, 32]. The research team revised and reworded the QUIPA tool following each focus group session before presenting the revised version to the subsequent group. Additional file 10 represents the final version of the QUIPA tool.

### Phase 2: Clinimetric evaluation of the QUIPA tool

The evaluation study was performed between August and December 2018. Participants with hip and/or knee OA were recruited to attend a single one-on-one consultation with a designated study physiotherapist for assessment and treatment of their affected joint(s). They were then required to complete the QUIPA tool online at three time points: one week (W1), twelve weeks (W12) and thirteen weeks (W13) after their consultation. A three-month recall period was selected for the QUIPA tool to capture either single or multi-session episodes of physiotherapy care and has been utilised in other comparable tools [22, 23]. For the purpose of this study, participants were asked not to have any further physiotherapy consultations for their affected hip and/or knee joint(s) during the thirteen weeks to avoid treatment confusion. For the purpose of this clinimetric evaluation, we also established a physiotherapist version of the QUIPA tool, which contained the same items but worded from the physiotherapists’ perspective. Physiotherapists completed the tool immediately post-consultation (W0). Ethical approval was granted by the School of Health Sciences Human Ethics Advisory Group, University of Melbourne (Ethics Application 1,750,925).

To evaluate patient test-retest reliability, we examined the participant responses between W12 and W13. We used three a priori hypotheses to assess construct validity. The hypotheses reflected anticipated response patterns among contrasting subgroups in relation to body mass index (BMI), pain level with walking and daily functional ability [21]. Criterion validity was determined by assessing agreement between physiotherapists and participants at W1. We defined responses from the physiotherapists as ‘gold standard’ as we expected their responses to be the most accurate compared to the participants since they completed the tool immediately after the consultation session and knew what treatment they had administered.

#### Study participants

A convenience sample of adults aged 45 years or over with self-reported hip and/or knee OA were recruited from the CHESM research database and by advertisements on Facebook. We aimed for a minimum of 50 people to participate in the clinimetric study because this sample size is the minimum recommended for any health questionnaire validity and/or reliability study [33]. The proposed minimum sample size allowed for a broad cross-sectional representation of people with hip and/or knee OA, including ages, genders and OA severity.

Participants were required to meet the National Institute for Health and Care Excellence OA clinical criteria: i) aged 45 years or above ii) have activity-related hip and/or knee pain and 3) have no more than 30 min of morning stiffness in their hip and/or knee. Participants were excluded if they had inflammatory arthritis, had undergone hip/knee replacement surgery for the affected hip/knee(s), planned to see another physiotherapist within thirteen weeks and/or were unable to give consent, attend an appointment with one of the study physiotherapists or to complete the questionnaires online at the specified time points.

We recruited nine physiotherapists currently registered to practise in Australia and working in private practice settings within Melbourne to ensure geographical spread around Melbourne for participants’ convenience.

#### Procedure

Participants received one consultation from their designated study physiotherapist at no cost to themselves. In order to increase variability in the care provided within a standard 30-min consultation, physiotherapists were provided with different cue cards that contained specific tasks/treatments they were requested to do, or not do, with the participants. Participants were informed that the physiotherapists were going to provide a range of different treatments to different participants, and thus individual participants did not have any pre-conceived ideas about what they would or would not receive. Participants were emailed a link to the online QUIPA tool at one, twelve and thirteen weeks following their physiotherapy session and were asked to complete the tool as soon as they could. With the W1 QUIPA tool, participants were also asked to provide information about demographic, other medical conditions, height and weight as well as to complete the pain and function subscales of the Western Ontario and McMaster Universities Osteoarthritis Index (WOMAC). Participants were asked whether they had seen another physiotherapist each time they completed the QUIPA tool. Reminder emails and text messages were sent to non-responders daily (up to three times) after responses were due. To maximise completion of surveys [34], those who completed all three were entered into a draw to win a $100 gift card.

Physiotherapists were asked to complete the QUIPA tool online immediately following each consultation. Physiotherapists were reimbursed $60 for each participant they saw.

### Statistical analysis

#### Reliability

Test-retest reliability of the QUIPA tool for participants was determined by comparing their responses between W12 (+/− 7 days) and W13 (− 2 /+ 7 days). Test-retest reliability for individual QI items was assessed by calculating Cohen’s Kappa (95% confidence intervals CI), percentage of observed agreement (i.e. the percentage of occasions when the answer was identical between W12 and W13), and percentage of expected agreement (i.e. the percentage of occasions when the answer was expected by chance to be identical between W12 and W13). Cohen’s Kappa compares the expected agreement to that observed. Kappa values were interpreted according to Landis and Koch [35]: 0–0.20 slight; 0.21–0.40 fair; 0.41–0.60 moderate; 0.61–0.80 substantial and 0.81–1.00 almost perfect reliability.

Test-retest for each QUIPA subscale and the total score was assessed with intraclass correlation coefficients (ICC) (95% CI) estimated using a two-way mixed effect model. An ICC of ≥0.70 was considered acceptable [33].

#### Validity

Construct validity was assessed with three predefined hypotheses. We first hypothesized that people responding ‘not overweight’ for the QI on benefits of losing weight (item #13a) would self-report lower BMI compared to those responding ‘yes’, ‘no’ or ‘don’t remember’. We also hypothesized that people responding ‘no such problems’ for the QIs on the walking aid item (#14) and the appliances and aids item (#15) would report no difficulty with walking and score lower for total physical function score on the WOMAC respectively compared to those responding ‘yes’, ‘no’ or ‘don’t remember’. Chi-square tests were used to test the first and second hypotheses and a t-test was used for the third hypothesis. The *p*-value cut off for statistical significance was ≤0.05 for both statistical tests. Validity was considered acceptable if ≥75% of the predefined hypotheses were confirmed [33].

Criterion validity of the QUIPA tool was determined by assessing agreement between physiotherapists and participants at W1 on individual items, each subscale and the total score of the QUIPA tool. To assess agreement for individual QI items, Cohen’s Kappa (95% CI), the percentage of observed agreement and percentage of expected agreement between physiotherapists and patients were calculated. Agreement for each subscale and the total score was assessed with an ICC (95% CI) estimated using a two-way mixed effect model.

#### Pass rates for individual QIs

The pass rate (%) for each QI was calculated based on responses from physiotherapists and patients at Week 1, where the numerator represented the total of ‘yes’ answers for the QI and the denominator was the total of ‘yes’ and ‘no’ answers for the QI. The denominator did not include other response options as they were deemed not relevant to a calculation of pass rate.

## Results

### Phase 1: tool development

#### Stage 1 – drafting of patient-reported quality indicators for physiotherapy care

Thirty recommendations were extracted from the consensus study that identified the clinical guideline recommendations most relevant to physiotherapy practice [28]. Of these, QIs were not developed for 11 recommendations and six recommendations were partly excluded (Additional file 2). The remaining 19 recommendations (Additional file 1) were converted into QIs, utilizing the phrasings from the UK-QI questionnaire [22] where possible. Of these 19, four recommendations were converted into two QIs each whilst only one QI was generated from each of the remaining 15 recommendations. Thus in total, 23 QIs formed the first draft of the QUIPA tool. Each QI was assigned with either a three or four-level response scale (i.e. ‘yes’/ ‘no’/ ‘don’t remember’ or ‘yes’/ ‘no’/ ‘don’t remember’/ ‘no such problems’ or ‘not overweight’ or ‘already doing own exercise program’) (Additional file 1).

### Stage 2 – refinement of the language and format of the QUIPA tool

#### Patient and public involvement

The first focus group was conducted with seven participants and the other two groups with four each. The mean (standard deviation) age of the participants was 63.9 (9.1) years and all had either knee OA or hip and knee OA. Participants’ characteristics are provided in Additional file 3.

#### Focus group feedback

Following feedback from the focus groups, several changes were made to the draft QUIPA tool (Additional file 1). This included reducing the number of items on the tool to ease participant burden (Q6-8a, Q17), removing items that were perceived to be too vague to participants (Q5 & 19), reducing words to improve clarity (Q1, 4, 8a, 8b, 8c, 9a, 9b, 16 and 18), avoiding multiple dimensions of care within a single item by splitting the QI into two questions (Q3) and expanding some QIs to improve specificity (Q2, 14). One item (Q20) was removed due to conflicting evidence supporting its effectiveness that emerged during the course of the study. Participants felt that the three-month recall period was appropriate and were satisfied with the response options and format of the tool.

#### Final QUIPA tool

The final version of the QUIPA tool comprised 18 items (Additional file 10: Table S1 and Additional file 1), organised into three subscales (Additional file 10: Table S1). The first subscale was Assessment and Management Planning and comprised the six items concerning OA assessment, comorbidities, screening for depression, depression referral, management planning and review. The second subscale was Core Recommended Treatments and contained the eight items concerning OA and related pain, education about different treatment options for OA, specific exercise program prescription, exercise preferences, exercise adherence, education about benefits of weight loss and strategies for losing weight. In this subscale, if ‘no’ was ticked for the item relating to specific exercise program prescription (item #10), then the item concerning exercise adherence (item #12) was automatically omitted by the scorer as not applicable. In addition, if an answer other than ‘yes’ was ticked for the item relating to benefits of weight loss (item #13a), the item addressing strategies for losing weight (item #13b) was also omitted as not applicable. The final subscale was Adjunctive Treatments and consisted of the four items relating to walking aids, appliances and aids, work-related advice and footwear.

#### Scoring instructions for the QUIPA tool

Table 1 represents the scoring instructions for the QUIPA tool. The pass rate (%) for each subscale was calculated independently, where the numerator represented the total of ‘yes’ ticked in the subscale and the denominator was the total of ‘yes’ and ‘no’ ticked in the subscale. For each subscale, if more than 50% of the items were not responded with ‘yes’ or ‘no’ answers, the response was considered invalid and the subscale score was not calculated. The total pass rate (%) of the QUIPA tool was calculated from all responses, where the numerator represented the total of ‘yes’ ticked on the tool, and the denominator was the total of ‘yes’ and ‘no’ ticked before the total score was normalized to 100. Percentage of score ranged from 0 to 100, with 100% representing the highest quality of care score.

**Table 1:** Scoring instructions for the Quality Indicators for Physiotherapy Management of Hip and Knee Osteoarthritis (QUIPA)

**Objective** The QUIPA tool is a patient-reported questionnaire used to assess patient perspectives on whether physiotherapists are providing evidence-based care when managing hip and/or knee osteoarthritis. It is a self-administered questionnaire consisting of 18 items divided into 3 subscales: • Assessment and management planning: Q1–6 • Core recommended treatments: Q7-13b • Adjunctive treatments: 14–17 The last 12 weeks is the time period considered when answering the questions.	
**Method of use** The QUIPA tool takes approximately 15 min to complete. For each question, tick ONLY **1** box corresponding to your response. For question **10**, if the answer was **‘no’**, question **12** should be omitted and not answered. For question **13a**, if the answer was **‘no’**, **‘don’t remember’** or **‘not overweight’,** question **13b** should be omitted and not answered.	
**Scoring instructions** Each subscale score is calculated independently as below. Do not score if there is a missing response or if the response is in the grey columns. For each subscale, if > 50% of the subscale items have not been responded with **‘yes’** or **‘no’** answers, the response is considered invalid and no subscale score should be calculated. For subscale Assessment and management planning, this means that 3 items must be answered; for Core recommended treatments, 4 items; and for Adjunctive treatments, 2 items must be answered in order to calculate a subscale score.	
**Score (S) for each subscale:** $\mathbf{S}=\frac{\mathbf{Total}\ \mathbf{of}`\mathbf{yes}'}{\mathbf{Total}\ \mathbf{of}`\mathbf{yes}'\&`\mathbf{no}'}\mathbf{X}\ \mathbf{100}\%$	
For the total score of the QUIPA tool, add up the responses from all 3 subscales as below. ** Total Score (ToS):** $\mathbf{ToS}=\frac{\mathbf{Total}\ \mathbf{of}`\mathbf{yes}'\mathbf{from}\ \mathbf{all}\ \mathbf{3}\ \mathbf{subscales}}{\mathbf{Total}\ \mathbf{of}`\mathbf{yes}'\&`\mathbf{no}'\mathbf{from}\ \mathbf{all}\ \mathbf{3}\ \mathbf{subscales}}\mathbf{X}\ \mathbf{100}\%$	
**Interpreting the results** The QUIPA tool is used to measure quality indicator pass rates for physiotherapists in managing hip/knee osteoarthritis. Percentage score is calculated, ranging from 0 to 100, with 100% representing the best quality of care score.	

### Phase 2: evaluation of the QUIPA tool

#### Characteristics of participants

Of 90 eligible participants, 65 (72%) attended a physiotherapy consultation session. More than half were female (63%) and the mean (standard deviation) age was 64.5 (8.1) years. The majority of the participants (80%) had only knee OA, 15% had hip and knee OA, and 5% had only hip OA (Additional file 4).

#### Characteristics of physiotherapists

Of the 16 physiotherapists who expressed interest in the project, nine (four female) were selected based on clinical practice locations. More than half of the physiotherapists had ≤10 years of clinical experience, worked clinically ≥31 h weekly and saw ≥10 patients with hip and/or knee OA monthly (Additional file 5).

#### Test-retest reliability

Of the 65 participants who attended physiotherapy consultations, 63 (97%) completed the QUIPA tool within the specified timeframes for W12 and W13. The Kappa coefficients for individual QI items ranged from 0.30–0.83, with one demonstrating ‘almost perfect’ agreement, one ‘substantial’, thirteen ‘moderate’ and three ‘fair’ agreement [35] (Table 2). The percentage of observed agreement ranged from 64 to 94% and expected agreement ranged from 30 to 64%. Of the 63 participants, 23 reported being ‘not overweight’. More than a third of the total participants selected ‘no such problems’ for QIs targeting OA subgroups i.e. item #14 walking aid (*n* = 23), item #15 appliances and aids (*n* = 32), item #16 work advice (*n* = 43) and item #3 depression screening (*n* = 38) at W12. The ICC (95% CI) for the Assessment and Management Planning subscale (*n* = 58) was 0.70 (0.54, 0.81), Core Recommended Treatments subscale (*n* = 56) was 0.84 (0.75, 0.90) and Adjunctive Treatments subscale (*n* = 20) was 0.70 (0.39, 0.87). The ICC (95% CI) for the total score (*n* = 63) was 0.80 (0.69, 0.88).

**Table 2:** Patient test-retest reliability for individual quality indicator at Week 12 and 13 (*n* = 63)

Quality Indicators ^**a**^	Cohen’s Kappa	95% CI	Observed agreement (%)	Expected agreement (%)
1. Osteoarthritis assessment	0.38	0.11, 0.62	77.8	64.3
2. Comorbidities	0.48	0.30, 0.65	68.3	38.6
3. Depression screening	0.48	0.27, 0.65	68.3	39.5
4. Depression referral	0.49	0.29, 0.70	76.2	53.3
5. Management plan	0.33	0.10, 0.53	66.7	50.2
6. Physiotherapy review	0.46	0.27, 0.63	71.4	47.3
7. Osteoarthritis definition	0.57	0.38, 0.74	74.6	41.6
8. Osteoarthritis pain	0.54	0.32, 0.72	76.2	48.7
9. Treatment risk and benefits	0.53	0.35, 0.71	71.4	39.7
10. Exercise prescription	0.83	0.67, 0.96	93.7	62.5
11. Exercise preference	0.30	0.10, 0.50	63.5	47.9
12. Exercise adherence (*n* = 60)	0.54	0.36, 0.70	70.0	35.0
13. a. Benefits of weight loss	0.77	0.64, 0.90	84.1	30.4
b. Strategies for weight loss (*n* = 19)	0.60	0.20, 0.90	78.9	47.4
14. Walking aid	0.47	0.28, 0.66	66.7	37.5
15. Appliances and aids	0.41	0.23, 0.58	66.7	43.8
16. Work advice	0.41	0.17, 0.62	77.8	62.4
17. Footwear advice	0.60	0.41, 0.75	74.6	36.3

95% CI: 95% confidence interval

*n* number of participants

^**a**^The complete quality indicator corresponding to each number can be found in Additional file 10 Table S1

#### Validity

##### Construct

Construct validity was considered acceptable with all three pre-defined hypotheses confirmed (Additional file 6).

##### Agreement between treating physiotherapists and participants at W1 -

The Kappa coefficients for individual QIs ranged from − 0.04-0.59, with two demonstrating ‘moderate’, eight demonstrating ‘fair’ and six demonstrating ‘slight’ agreement [35]. The Kappa coefficients for two QIs were below 0 (Table 3). The percentage of observed agreement ranged from 46 to 86% and for expected agreement from 32 to 75%. The ICC (95% CI) for the Assessment and Management Planning subscale (*n* = 63) was 0.20 (− 0.05, 0.43), Core Recommended Treatments subscale (*n* = 65) was 0.06 (− 0.19, 0.29) and Adjunctive Treatments subscale (*n* = 21) was 0.70 (0.39, 0.87). The ICC (95% CI) for the total score (*n* = 65) was 0.11 (− 0.14, 0.34).

**Table 3:** Agreement between treating physiotherapists and participants (*n* = 65) at Week 1 for individual quality indicator

Quality Indicators ^**a**^	Cohen’s Kappa	95% CI	Observed agreement (%)	Expected agreement (%)
1. Osteoarthritis assessment	0.09	−0.06-0.24	76.9	74.6
2. Comorbidities	0.17	−0.01-0.38	61.5	53.5
3. Depression screening	0.21	0.04–0.40	69.2	61.1
4. Depression referral	0.26	0–0.50	64.6	52.0
5. Management plan	−0.01	−0.16-0.18	67.7	68.0
6. Physiotherapy review	0.10	−0.06-0.27	46.2	39.9
7. Osteoarthritis definition	0.36	0.12–0.59	73.8	59.0
8. Osteoarthritis pain	0.25	0.03–0.46	66.2	54.6
9. Treatment risk and benefits	−0.04	−0.23-0.16	46.2	48.1
10. Exercise prescription	0.29	0.07–0.48	75.4	65.4
11. Exercise preference	0.09	−0.12-0.32	72.3	69.5
12. Exercise adherence (*n* = 54)	0.23	−0.02-0.45	59.3	47.2
13. a. Benefits of weight loss	0.30	0.13–0.46	52.3	32.1
b. Strategies for weight loss (*n* = 14)	0.59	0–1	85.7	65.7
14. Walking aid	0.37	0.17–0.55	61.5	39.1
15. Appliances and aids	0.18	−0.05-0.39	58.5	49.6
16. Work advice	0.05	−0.17-0.28	63.1	61.3
17. Footwear advice	0.41	0.17–0.61	70.8	50.7

95% CI: 95% confidence interval

*n* number of participants

^**a**^The complete quality indicator corresponding to each number can be found in Additional file 10 Table S1

##### Pass rates for individual QIs

Additional file 7and 8 show the pass rates for individual QIs as reported by physiotherapists and patients at Week 1 respectively.

## Discussion

This study developed a patient-reported QI tool to measure and benchmark physiotherapy care for people with hip and/or knee OA. A clinimetric evaluation of the QI tool was then performed to establish its reliability and validity in assessing physiotherapy care for this patient group.

Test-retest reliability for each subscale and total score of the QUIPA tool was acceptable (ICC of ≥0.70) although in most cases, the lower bound of the CIs was below 0.70, reflecting variability in the data and/or limited sample size. However, reliability for individual items varied. The item on exercise prescription (item #10) was the only QI that achieved ‘almost perfect’ agreement while the item relating to discussing the benefits of weight loss (item #13a) reached ‘substantial’ agreement. The better reliability of these two items compared to that of others suggests that it was easier for patients to understand their intent and to recall whether or not these aspects of physiotherapy care were provided.

Most of the other items (*n* = 13) achieved ‘moderate’ agreement with three attaining ‘fair’ agreement. Ten achieved high observed agreement (> 70%) despite high variability in their Kappa estimates (as indicated by CI). This may be due to the statistical effect of a high or low prevalence of a specific answer for those items. High or low prevalence reduces Kappa estimates despite high observed agreement [36, 37]. For example, for the QI related to OA assessment (item #1), despite the high observed agreement (78%), the Kappa estimate (95% CI) =0.38 (0.11, 0.62) is low due to high prevalence of ‘yes’ (53 out of 63) responses (leading to a high expected agreement). If agreement is expected to be high by chance, perhaps because most participants select the same value for an item, then even if observed agreement is high, Cohen’s Kappa will be low. Conversely, for the QI related to OA pain (item #8), despite the observed agreement (76%) being comparable to item #1, the prevalence of ‘yes’ (34 out of 63) response resulted in a higher Kappa estimate (95% CI) =0.54 (0.32, 0.72) (Additional file 9). The three items with the lowest Kappa estimates were related to OA assessment, management plan and exercise preference. Despite efforts to maximise specificity, these items likely remained ambiguous and could be interpreted differently across participants. Another potential reason for disagreement between test and retest scores was related to poor recall as we observed interchanges between “yes/no” and “don’t remember” response options within an individual at W12 and W13. We deliberately chose a 3-month window when developing the QUIPA tool in order to capture multi-session episodes of physiotherapy care. Thus, we evaluated test-retest reliability of the tool at thirteen weeks, the period of maximum recall, in order to establish reliability in the ‘worst case’ scenario. Reliability may be greater with shorter recall periods.

Overall, despite generally low Kappa values for single items of the QUIPA tool, the test-retest Kappa estimates and observed agreement were comparable [21] or only slightly lower [30] than previous patient-reported QI tools for OA care which have been rolled out and now used in practice. However, it must be noted that these studies used a recall time frame of 2 weeks for evaluating test-retest reliability despite the tools having a maximum recall period of 3 months [21, 30]. It is therefore not known whether the reliability estimates they reported would have been lower if they had used a three-month recall as we did.

In terms of validity, the QUIPA tool has acceptable construct validity with all three pre-defined hypotheses confirmed (*P* < 0.05). These hypotheses were similar to those used for assessment of construct validity in other QI tools for OA care [21, 30], although the sample size in our subgroups was smaller. While construct validity was supported, our data indicate that the tool does not have acceptable criterion validity as assessed via comparison of participants’ responses at W1 to responses provided by the physiotherapists.

The subscale scores, total scores and most of the individual items of the QUIPA tool achieved low agreement between participants and physiotherapists. Although the recall period for participants was shorter for validity testing, it is possible that treating physiotherapists might have delivered the care as described by the QIs, but participants might not remember receiving the care or misinterpreted the care received. Despite the consumer input to the development of the QI items, it appears that some items were ambiguous and likely to be interpreted differently, particularly from the perspective of a patient or a clinician. For example, for the item relating to review (item #6), a treating physiotherapist might suggest the participant see a physiotherapist for their hip and/or knee OA only when their symptoms flared up and would select the ‘yes’ response to this QI. For the participants, they might only select the ‘yes’ response if their treating physiotherapist proposed a specific date for their next physiotherapy review. It was also not clear for clinicians as to which responses to select if the participant voluntarily offered information relating to certain QIs without any promptings from their treating physiotherapist. Finally, for QIs that were not applicable to all participants (e.g. benefits of weight loss, walking aid, appliances and aids, work advice and depression referral), there were large inconsistencies between participants and their treating physiotherapists concerning whether the ‘no’ or the ‘not overweight’ / ‘no such problems’ option was selected. For the item relating to discussing the benefits of weight loss (item #13a), perceptions of overweight/obese can also differ between the participant and their physiotherapist. Overall, it appears difficult to generate items that are unambiguous, interpreted in the same way by different users and capture all variations in provision of care.

### Future directions

This study lays the groundwork for future refinement of the QUIPA tool, a patient-reported QI for benchmarking quality of physiotherapy care in hip and/or knee OA. Further refinement and re-evaluation are required to improve the validity of the QUIPA tool. Considerations for future refinements include a patient recall period shorter than 3 months, removal of ambiguous items, development of more comprehensive instructions to patients about what they should consider when answering the items and reduction of response options.

### Strengths and limitations

This study has several strengths. The QIs were generated from an international physiotherapy consensus exercise [28] that used high-quality clinical guidelines for hip and knee OA [1, 29]. Other strengths include robust methodology to develop QIs (e.g. defined scope and purpose of the QIs, involvement of patients and physiotherapists, formulation of specific and measurable QIs [16, 38]) and good response rates to all surveys. In addition, no previous studies have comprehensively evaluated the validity of patient-reported QIs by assessing agreement between patients and their treating clinicians.

Despite achieving the recommended sample size for clinimetric testing, there was limited variation in the profiles of the participants. As such, we had few participants within subgroups such as those who were overweight, had problems with their walking, daily activities or work due to OA and with depression. Given these small sample sizes, and the aim of this work, we elected not to adjust for patient characteristics. Doing so may introduce bias and noise into our estimates of interest. In addition, during the course of this study, we were made aware of the use of pre-treatment ‘registration’ forms in some physiotherapy clinics, which may contain questions relating to the QIs. If information is collected via a form before a consultation rather than via a discussion during a consultation, this may lead to difficulties deciding how to answer the QUIPA items. Finally, despite attempts to increase variability in the data, the majority of the participants and treating physiotherapists chose ‘yes’ as their response options to the QIs.

## Conclusion

In conclusion, this study developed the first patient-reported QI tool specifically to evaluate physiotherapy care for hip and/or knee OA. The QUIPA tool showed acceptable test-retest reliability for subscales and total score but inadequate reliability for individual items. Construct validity was confirmed but criterion validity for individual items, subscales and the total score was inadequate. Further research is needed to refine the QUIPA tool to improve its clinimetric properties before it can be used to accurately assess quality of physiotherapy care for hip and/or knee OA.

## Supplementary information

**Additional file 1.** The development of the Quality Indicators for Physiotherapy Management of Hip and Knee Osteoarthritis (QUIPA) tool.

**Additional file 2.** Excluded recommendations.

**Additional file 3.** Characteristics of participants in the focus groups.

**Additional file 4.** Characteristics of participants in the validation study.

**Additional file 5.** Characteristics of physiotherapists in the validation study.

**Additional file 6.** Construct validity analyses based on three predefined hypotheses.

**Additional file 7.** Pass rates for individual quality indicators reported by physiotherapists.

**Additional file 8.** Pass rates for individual quality indicators reported by patients at Week 1.

**Additional file 9.** Quality indicator example for statistical prevalence.

**Additional file 10.** The Quality Indicators for Physiotherapy Management of Hip and Knee Osteoarthritis (QUIPA).

## Data Availability

All data generated or analysed during this study are included in this published article [and its supplementary information files].

## Abbreviations

OA: Osteoarthritis
QUIPA: Quality Indicators for Physiotherapy Management of Hip and Knee Osteoarthritis
ICC: Intraclass correlation coefficient
QIs: Quality Indicators
UK: United Kingdom
W1: Week one
W12: Week twelve
W13: Week thirteen
W0: Post-consultation
BMI: Body mass index
WOMAC: Western Ontario and McMaster Universities Osteoarthritis Index
