# Supplementary material for: Patient-reported quality indicators to evaluate physiotherapy care for hip and/or knee osteoarthritis- development and evaluation of the QUIPA tool
Source: BMC Musculoskelet Disord. 2020 Apr 1;21:202. doi: 10.1186/s12891-020-03221-5 (PMC7114805; doi:10.1186/s12891-020-03221-5)
Supplement: Supplementary file 1 — Additional file 1. The development of the Quality Indicators for Physiotherapy Management of Hip and Knee Osteoarthritis (QUIPA) tool. [file 12891_2020_3221_MOESM1_ESM.zip › Additional file 1.docx]

**Additional file 1:** The development of the Quality Indicators for Physiotherapy Management of Hip and Knee Osteoarthritis (QUIPA) tool. The QUIPA tool was originally generated from the final list of ranked recommendations in a consensus study [28], compared with the United Kingdom-Quality Indicator (UK-QI) questionnaire [22] and later transformed into question format by utilizing some of the wordings from the UK-QI questionnaire (Stage 1) before being refined further in the focus groups (Stage 2).
