# Supplementary material for: Patient-reported quality indicators to evaluate physiotherapy care for hip and/or knee osteoarthritis- development and evaluation of the QUIPA tool
Source: BMC Musculoskelet Disord. 2020 Apr 1;21:202. doi: 10.1186/s12891-020-03221-5 (PMC7114805; doi:10.1186/s12891-020-03221-5)
Supplement: Supplementary file 2 — Additional file 2. Excluded recommendations. [file 12891_2020_3221_MOESM2_ESM.zip › Additional file 2.docx]

**Additional file 2:** These recommendations from the consensus study [28] that identified the clinical guideline recommendations most relevant to physiotherapy practice were not used to develop quality indicators for physiotherapy management of hip and knee osteoarthritis.
