# Supplementary material for: Patient-reported quality indicators to evaluate physiotherapy care for hip and/or knee osteoarthritis- development and evaluation of the QUIPA tool
Source: BMC Musculoskelet Disord. 2020 Apr 1;21:202. doi: 10.1186/s12891-020-03221-5 (PMC7114805; doi:10.1186/s12891-020-03221-5)
Supplement: Supplementary file 3 — Additional file 3. Characteristics of participants in the focus groups. [file 12891_2020_3221_MOESM3_ESM.docx]

**Additional file 3:** Characteristics of participants in the focus groups (n=15)

|  | **Mean (SD) or n (%)** |
| --- | --- |
| **Female** | 12 (80%) |
| **Age** | 63.9 (9.1) |
| **Education** |  |
| -Less than three years of high school | 1 (7%) |
| -Three years or more of high school | 1 (7%) |
| -Some tertiary training | 4 (27%) |
| -Graduated from university or polytechnic | 2 (13%) |
| -Any post-graduate study | 7 (47%) |
| **Joint(s) affected by osteoarthritis** |  |
| -Hip | 0 (0%) |
| -Knee | 8 (53%) |
| -Hip and knee | 7 (47%) |
| **Knee pain (NRS)** | 4.9 (2.2) |
| **Restriction to daily activities (NRS)** | 3.4 (2.2) |

SD: standard deviation

n: number of participants

NRS: numeric rating scale; ranges from 0 to 10 where lower scores indicate less pain or restriction to daily activities
