# Supplementary material for: Patient-reported quality indicators to evaluate physiotherapy care for hip and/or knee osteoarthritis- development and evaluation of the QUIPA tool
Source: BMC Musculoskelet Disord. 2020 Apr 1;21:202. doi: 10.1186/s12891-020-03221-5 (PMC7114805; doi:10.1186/s12891-020-03221-5)
Supplement: Supplementary file 4 — Additional file 4. Characteristics of participants in the validation study. [file 12891_2020_3221_MOESM4_ESM.docx]

**Additional file 4:** Characteristics of participants in the validation study (n=65)

|  | **Mean (SD) or n (%)** |
| --- | --- |
| **Female** | 41 (63%) |
| **Age** | 64.5 (8.1) |
| **Body mass index (kg/m^2^)** | 29.6 (6.5) |
| **Comorbidity** |  |
| -Had one other chronic disease ^a^ | 7 (11%) |
| -No other chronic diseases | 58 (89%) |
| **Education** |  |
| -Less than three years of high school | 1 (2%) |
| -Three years or more of high school | 7 (11%) |
| -Some tertiary training | 15 (23%) |
| -Graduated from university or polytechnic | 26 (40%) |
| -Any post-graduate study | 16 (25%) |
| **Joint(s) affected by osteoarthritis (self-reported)** |  |
| -Hip | 3 (5%) |
| -Knee | 52 (80%) |
| -Hip and knee | 10 (15%) |
| **Pain (WOMAC)** | 5.9 (3.0) |
| **Physical function (WOMAC)** | 35.5 (11.0) |
|  |  |
| SD: standard deviation |  |
| n: number of participants  WOMAC: Western Ontario and McMaster Universities Osteoarthritis Index; pain subscale ranges from 0 to 20 and physical function subscale ranges from 0 to 68, where lower scores indicate less pain and better function respectively  ^a^Included diabetes (n=4), tumour (n=2), leukemia (n=1) | |
