# Supplementary material for: Patient-reported quality indicators to evaluate physiotherapy care for hip and/or knee osteoarthritis- development and evaluation of the QUIPA tool
Source: BMC Musculoskelet Disord. 2020 Apr 1;21:202. doi: 10.1186/s12891-020-03221-5 (PMC7114805; doi:10.1186/s12891-020-03221-5)
Supplement: Supplementary file 5 — Additional file 5. Characteristics of physiotherapists in the validation study. [file 12891_2020_3221_MOESM5_ESM.docx]

**Additional file 5:** Characteristics of physiotherapists in the validation study (n=9)

|  | **Mean (SD) or n (%)** |
| --- | --- |
| **Female** | 4 (44%) |
| **Age** | 34.3 (10.1) |
| **Number of years in clinical practice** |  |
| ≤ 10 years | 5 (56%) |
| 11-20 years | 3 (33%) |
| 31-40 years | 1 (11%) |
| **Average number of clinical practice hours weekly** |  |
| 11-20 hours | 2 (22%) |
| 21-30 hours | 1 (11%) |
| 31 hours and above | 6 (67%) |
| **Average number of patients with hip and/or knee osteoarthritis seen monthly** |  |
| ≤ 5 people | 3 (33%) |
| 6- 9 people | 1 (11%) |
| ≥ 10 people | 5 (56%) |
| SD: standard deviation |  |
| n: number of participants |  |
|  | |
