# Supplementary material for: Patient-reported quality indicators to evaluate physiotherapy care for hip and/or knee osteoarthritis- development and evaluation of the QUIPA tool
Source: BMC Musculoskelet Disord. 2020 Apr 1;21:202. doi: 10.1186/s12891-020-03221-5 (PMC7114805; doi:10.1186/s12891-020-03221-5)
Supplement: Supplementary file 6 — Additional file 6. Construct validity analyses based on three predefined hypotheses. [file 12891_2020_3221_MOESM6_ESM.docx]

**Additional file 6:** Construct validity analyses based on three predefined hypotheses

| **Hypotheses (based on patient week one data)** | **Analysis Value** | **Hypothesis confirmed?** |
| --- | --- | --- |
| 1. People responding ‘not overweight’ on item #13a are more likely to report BMI < 25 (not overweight) than people responding ‘yes’, ‘no’ or ‘don’t remember’ | t = 22.1 P < 0.001  12 (selected ‘not overweight’) versus 2 (selected ‘yes’, ‘no’ or ‘don’t remember’) reporting BMI < 25  n = 20 versus 45 | Yes |
| 1. People responding ‘no such problems’ on item #14 are likely to score ‘0’ with walking item (WOMAC item 11 -walking on a flat surface) than people responding ‘yes’, ‘no’ or ‘don’t remember’ | t = 7.5 P < 0.01  n = 16 (selected ‘no such problems’) versus 5 (selected ‘yes’, ‘no’ or ‘don’t remember’) scoring ‘0’ with walking item  n = 32 versus 33 | Yes |
| 1. People responding ‘no such problems’ on item #15 have on average a lower total physical function score for WOMAC than people responding ‘yes’, ‘no’ or ‘don’t remember’ | Mean value (SD): 33.1 (9.8) vs 39.2 (11.8) ^a^  Mean difference (95% CI):  6.1 (0.7, 11.5) P < 0.05  n = 39 (selected ‘no such problems’) versus 26 (selected ‘yes’, ‘no’ or ‘don’t remember’) | Yes |

BMI: Body mass index (kg/m^2^)

QI: Quality Indicator in the Quality Indicators for Physiotherapy Management of Hip and Knee Osteoarthritis (QUIPA) tool. The complete quality indicator corresponding to each number can be found in Table 1.

WOMAC: Western Ontario and McMaster Universities Osteoarthritis Index; walking item ranges from 0 to 4, where lower score indicates lesser difficulty with walking

n: number of participants

P: probability value

Chi-square test (t)

^a^ Independent T-test
