# Supplementary material for: Patient-reported quality indicators to evaluate physiotherapy care for hip and/or knee osteoarthritis- development and evaluation of the QUIPA tool
Source: BMC Musculoskelet Disord. 2020 Apr 1;21:202. doi: 10.1186/s12891-020-03221-5 (PMC7114805; doi:10.1186/s12891-020-03221-5)
Supplement: Supplementary file 7 — Additional file 7. Pass rates for individual quality indicators reported by physiotherapists. [file 12891_2020_3221_MOESM7_ESM.docx]

**Additional file 7:** Pass rates for individual quality indicators reported by physiotherapists (n=65)

| **Quality Indicators ^a^** | **Other answers ^b^ except ‘yes’ and ‘no’** | **Missing data** | **Eligible persons ^c^** | **Quality Indicator pass rates ^d^** | |
| --- | --- | --- | --- | --- | --- |
|  |  |  |  | ‘Yes’ (%) | 95% CI |
| 1. Osteoarthritis assessment | - | - | 65 | 57 (87.7%) | 80-95 |
| 1. Comorbidities | 2 | - | 63 | 42 (66.7%) | 56-79 |
| 1. Depression screening | - | - | 65 | 8 (12.3%) | 5-22 |
| 1. Depression referral | 41 | - | 24 | 4 (16.7%) | 4-33 |
| 1. Management plan | - | - | 65 | 58 (89.2%) | 82-95 |
| 1. Physiotherapy review | 1 | - | 64 | 41 (64.1%) | 52-75 |
| 1. Osteoarthritis definition | - | - | 65 | 48 (73.8%) | 63-85 |
| 1. Osteoarthritis pain | - | - | 65 | 43 (66.2%) | 54-79 |
| 1. Treatment risk & benefits | 1 | - | 64 | 39 (60.9%) | 48-72 |
| 1. Exercise prescription | 6 | - | 59 | 49 (83.1%) | 73-92 |
| 1. Exercise preference | - | - | 65 | 56 (86.2%) | 77-94 |
| 1. Exercise adherence | - | 10 | 55 | 33 (60.0%) | 46-73 |
| 1. a. Benefits of weight loss | 32 | - | 33 | 19 (57.6%) | 39-73 |
| b. Strategies for weight loss | 1 | 46 | 18 | 12 (66.7%) | 44-89 |
| 1. Walking aid | 39 | - | 26 | 13 (50.0%) | 31-69 |
| 1. Appliances and aids | 46 | - | 19 | 2 (10.5%) | 0-26 |
| 1. Work advice | 51 | - | 14 | 5 (35.7%) | 14-64 |
| 1. Footwear advice | - | - | 65 | 24 (36.9%) | 25-49 |

95% CI: 95% confidence interval

n: number of participants

**^a^**The complete quality indicator corresponding to each number can be found in Table 1.

**^b^** Don’t remember/No such problems/ Already doing own exercise program/ Don’t have an exercise program/ Not overweight/ not employed

^c^ Total study sample minus missing data/ Don’t remember/No such problems/ Already doing own exercise program/ Don’t have an exercise program/ Not overweight/ not employed

^d^ Eligible persons reporting ‘yes’
