# Supplementary material for: Patient-reported quality indicators to evaluate physiotherapy care for hip and/or knee osteoarthritis- development and evaluation of the QUIPA tool
Source: BMC Musculoskelet Disord. 2020 Apr 1;21:202. doi: 10.1186/s12891-020-03221-5 (PMC7114805; doi:10.1186/s12891-020-03221-5)
Supplement: Supplementary file 8 — Additional file 8. Pass rates for individual quality indicators reported by patients at Week 1. [file 12891_2020_3221_MOESM8_ESM.docx]

**Additional file 8:** Pass rates for individual quality indicators reported by patients at Week 1 (n=65)

| **Quality Indicators ^a^** | **Other answers ^b^ except ‘yes’ and ‘no’** | **Missing data** | **Eligible persons ^c^** | **Quality Indicator pass rates ^d^** | |
| --- | --- | --- | --- | --- | --- |
|  |  |  |  | ‘Yes’ (%) | 95% CI |
| 1. Osteoarthritis assessment | 8 | - | 57 | 55 (96.5%) | 91-100 |
| 1. Comorbidities | 6 | - | 59 | 48 (81.4%) | 71-92 |
| 1. Depression screening | 12 | - | 53 | 9 (17.0%) | 8-28 |
| 1. Depression referral | 45 | - | 20 | 3 (15.0%) | 0-30 |
| 1. Management plan | 4 | - | 61 | 48 (78.7%) | 67-89 |
| 1. Physiotherapy review | 6 | - | 59 | 18 (30.5%) | 19-42 |
| 1. Osteoarthritis definition | 4 | - | 61 | 47 (77.0%) | 66-87 |
| 1. Osteoarthritis pain | 5 | - | 60 | 47 (78.3%) | 67-88 |
| 1. Treatment risk and benefits | 7 | - | 58 | 41 (70.7%) | 59-81 |
| 1. Exercise prescription | 8 | - | 57 | 55 (96.5%) | 91-100 |
| 1. Exercise preference | 5 | - | 60 | 51 (85.0%) | 75-93 |
| 1. Exercise adherence | 7 | 2 | 56 | 33 (58.9%) | 46-73 |
| 1. a. Benefits of weight loss | 21 | - | 44 | 20 (45.5%) | 32-61 |
| b. Strategies for weight loss | - | 45 | 20 | 15 (75.0%) | 55-95 |
| 1. Walking aid | 34 | - | 31 | 15 (48.4%) | 29-65 |
| 1. Appliances and aids | 41 | - | 24 | 7 (29.2%) | 13-50 |
| 1. Work advice | 48 | - | 17 | 3 (17.6%) | 0-35 |
| 1. Footwear advice | 4 | - | 61 | 21 (34.4%) | 23-46 |

95% CI: 95% confidence interval

n: number of participants

**^a^**The complete quality indicator corresponding to each number can be found in Table 1.

**^b^** Don’t remember/No such problems/ Already doing own exercise program/ Don’t have an exercise program/ Not overweight/ not employed

^c^ Total study sample minus missing data/ Don’t remember/No such problems/ Already doing own exercise program/ Don’t have an exercise program/ Not overweight/ not employed

^d^ Eligible persons reporting ‘yes’
