# Supplementary material for: Patient-reported quality indicators to evaluate physiotherapy care for hip and/or knee osteoarthritis- development and evaluation of the QUIPA tool
Source: BMC Musculoskelet Disord. 2020 Apr 1;21:202. doi: 10.1186/s12891-020-03221-5 (PMC7114805; doi:10.1186/s12891-020-03221-5)
Supplement: Supplementary file 9 — Additional file 9. Quality indicator example for statistical prevalence. [file 12891_2020_3221_MOESM9_ESM.docx]

**Additional file 9:** Frequency of ‘yes’, ‘no’ and ‘don’t remember’ responses for QI on osteoarthritis assessment (item #1) compared to QI for osteoarthritis pain (item #8) at Week 12 and 13.

| Item #1: osteoarthritis assessment | |  | Week 13 |  |  | Item #8: osteoarthritis pain | |  | Week 13 |  |
| --- | --- | --- | --- | --- | --- | --- | --- | --- | --- | --- |
|  |  | Yes | No | Don’t remember |  |  |  | Yes | No | Don’t remember |
|  | Yes | 43 | 3 | 3 |  |  | Yes | 34 | 4 | 3 |
| Week 12 | No | 4 | 5 | 0 |  | Week 12 | No | 2 | 10 | 0 |
|  | Don’t remember | 3 | 1 | 1 |  |  | Don’t remember | 5 | 1 | 4 |

QI = Quality Indicator in the Quality Indicators for Physiotherapy Management of Hip and Knee Osteoarthritis (QUIPA) tool. The complete quality indicator corresponding to each number can be found in Table 1.
