# Supplementary material for: Patient-reported quality indicators to evaluate physiotherapy care for hip and/or knee osteoarthritis- development and evaluation of the QUIPA tool
Source: BMC Musculoskelet Disord. 2020 Apr 1;21:202. doi: 10.1186/s12891-020-03221-5 (PMC7114805; doi:10.1186/s12891-020-03221-5)
Supplement: Supplementary file 10 — Additional file 10. The Quality Indicators for Physiotherapy Management of Hip and Knee Osteoarthritis (QUIPA). [file 12891_2020_3221_MOESM10_ESM.docx]

Table S1 The Quality Indicators for Physiotherapy Management of Hip and Knee Osteoarthritis (QUIPA)

| • We would like to know what treatment, information or advice you have received from our study physiotherapist for your **hip and/or knee osteoarthritis** **only.**  • For each question, please put a cross in one of the boxes provided. | | | | | |
| --- | --- | --- | --- | --- | --- |
| **Assessment and management planning** | | | | | |
|  | | **Yes** | **No** | **Don’t remember** | |
| 1 | Did a physiotherapist ask about how your osteoarthritis affects your life (such as your function, occupation, relationships and/or leisure activities)? |  |  |  | |
| 2 | Did a physiotherapist ask about any other medical conditions (such as heart problem or diabetes)? |  |  |  | |
| 3 | Did a physiotherapist ask if you are feeling anxious, stressed, down, depressed and/or have lost interest in activities you previously enjoyed? |  |  |  | |
|  | | **Yes** | **No** | **Don’t remember** | **No such problems** |
| 4 | If you are feeling anxious, stressed, down, depressed and/or have lost interest in activities you previously enjoyed, did a physiotherapist offer you a referral to another health professional who may be able to assist you with this? |  |  |  |  |
|  | | **Yes** | **No** | **Don’t remember** | |
| 5 | Did you discuss and agree with a physiotherapist on your management plan for your osteoarthritis? |  |  |  | |
| 6 | Did a physiotherapist discuss and suggest when you should seek another physiotherapy appointment for your osteoarthritis? |  |  |  | |
| **Total of ‘yes’ responses** | |  | | | |
| **Total of ‘no’ responses** | |  | | | |
| **Total of ‘yes’ & ‘no’ responses** | |  | | | |
| **(Total of ‘yes’ / total of ‘yes’ & ‘no’) X 100%** | |  | | | |
| **Core recommended treatments** | | | | | |
|  | | **Yes** | **No** | **Don’t remember** | |
| 7 | Did a physiotherapist discuss what osteoarthritis is? |  |  |  | |
| 8 | Did a physiotherapist discuss how pain may be caused by osteoarthritis? |  |  |  | |
| 9 | Did a physiotherapist discuss different treatment options available to manage your osteoarthritis including the risk and benefits involved? |  |  |  | |
|  | | **Yes** | **No** | **Don’t remember** | **Already doing own exercise program** |
| 10 | Did a physiotherapist offer you a specific exercise program for you to perform regularly for your osteoarthritis?  *(If you answered ‘no’ for this question, skip question 12)* |  |  |  |  |
|  | | **Yes** | **No** | **Don’t remember** | |
| 11 | Did a physiotherapist consider your personal situation and preferences when discussing a specific exercise program? |  |  |  | |
|  |  | **Yes** | **No** | **Don’t remember** | **Don’t have an exercise program** |
| 12 | Did a physiotherapist discuss ways to help you stick to a specific exercise program? |  |  |  |  |
|  | | **Yes** | **No** | **Don’t remember** | **Not overweight** |
| 13a | If you are overweight, did a physiotherapist discuss the benefits of losing weight?  *(If you answered ‘no/don’t remember/not overweight’ for this question, go to question 14)* |  |  |  |  |
|  | | **Yes** | **No** | **Don’t remember** | |
| 13b | If you answered ‘yes’ to question 13a, did a physiotherapist discuss ways to lose weight (such as seeing a dietician or joining a weight-loss group)? |  |  |  | |
| **Total of ‘yes’ responses** | |  | | | |
| **Total of ‘no’ responses** | |  | | | |
| **Total of ‘yes’ & ‘no’ responses** | |  | | | |
| **(Total of ‘yes’ / total of ‘yes’ & ‘no’) X 100%** | |  | | | |
| **Adjunctive treatments** | | | | | |
|  | | **Yes** | **No** | **Don’t remember** | **No such problems** |
| 14 | If you have difficulty with walking, did a physiotherapist discuss the option of a walking aid (such as stick, crutch or walker)? |  |  |  |  |
| 15 | If you have problems with daily activities, did a physiotherapist discuss the option of appliances and aids (such as assistive devices for personal hygiene, for example a special chair to assist you when showering) or offer you a referral to another health professional who may be able to assist with this (such as occupational therapist)? |  |  |  |  |
|  | | **Yes** | **No** | **Don’t remember** | **No such problems/ not employed** |
| 16 | If you are employed and have problems with performing your job due to your osteoarthritis, did a physiotherapist offer you any work-related advice or a referral to another health professional or service that may be able to assist with this (such as occupational rehabilitation)? |  |  |  |  |
|  | | **Yes** | **No** | **Don’t remember** | |
| 17 | Did a physiotherapist offer you advice on appropriate and comfortable footwear? |  |  |  | |
| **Total of ‘yes’ responses** | | | | | |
| **Total of ‘no’ responses** | | | | | |
| **Total of ‘yes’ & ‘no’ responses** | | | | | |
| **(Total of ‘yes’ / total of ‘yes’ & ‘no’) X 100%** | | | | | |
| **For the Total scoring, add up responses from all 3 subscales** | | | | | |
| **Total of ‘yes’ responses** | | | | | |
| **Total of ‘no’ responses** | | | | | |
| **Total of ‘yes’ & ‘no’ responses** | | | | | |
| **(Total of ‘yes’ / total of ‘yes’ & ‘no’) X 100%** | | | | | |
